# Elementary school teachers’ perspectives about learning during the COVID-19 pandemic

**DOI:** 10.1038/s41539-023-00191-w

**Published:** 2023-09-18

**Authors:** Aymee Alvarez-Rivero, Candice Odgers, Daniel Ansari

**Affiliations:** 1 Department of Psychology, University of Western Ontario, London, ON Canada; 2 Department of Psychological Science, University of California -Irvine, Irvine, CA USA; 3 Social Science Research Institute, Duke University, Durham, NC USA

**Keywords:** Education, Society

## Abstract

How did school closures affect student access to education and learning rates during the COVID-19 pandemic? How did teachers adapt to the new instructional contexts? To answer these questions, we distributed an online survey to Elementary School teachers (*N* = 911) in the United States and Canada at the end of the 2020–2021 school year. Around 85.8% of participants engaged in remote instruction, and nearly half had no previous experience teaching online. Overall, this transition was challenging for most teachers and more than 50% considered they were not as effective in the classroom during remote instruction and reported not being able to deliver all the curriculum expected for their grade. Despite the widespread access to digital technologies in our sample, nearly 65% of teachers observed a drop in class attendance. More than 50% of participants observed a decline in students’ academic performance, a growth in the gaps between low and high-performing students, and predicted long-term adverse effects. We also observed consistent effects of SES in teachers’ reports. The proportion of teachers reporting a drop in performance increases from 40% in classrooms with high-income students, to more than 70% in classrooms with low-income students. Students in lower-income households were almost twice less likely to have teachers with previous experience teaching online and almost twice less likely to receive support from adults with homeschooling. Overall, our data suggest the effects of the pandemic were not equally distributed.

## Introduction

The sudden onset of the COVID-19 pandemic had a profound effect on education worldwide^1,2^, with the aftermath of more than 180 countries experiencing school closures and more than 1.5 billion students left out of school^3^. Despite the efforts of governments and education institutions to provide alternative learning opportunities, the long periods that students had to spend away from the classroom have raised concerns about the potential long-term consequences on academic achievement, and the unequal effect that it will have on students from vulnerable and marginalized groups^4^, who had to navigate the challenges of at-home schooling while their families struggled with financial burdens^5^.

Empirical data about changes in students’ performance has been slow to emerge. One of the earliest pieces of evidence comes from a study in The Netherlands by Engzell, Frey, and Verhagen^6^. The authors analyzed changes in performance associated with school closures, using a uniquely rich dataset with more than 350,000 students in primary school. The data included biannual test scores collected at the middle and the end of each school year from 2017 and 2020. Critically, in 2020, the mid-year tests took place right before the first school closures in The Netherlands, providing a benchmark that authors could use to estimate learning losses. The authors identified an overall decrease in academic performance equivalent to 0.08 standard deviation units. Moreover, the effects on learning outcomes were not uniform, as students from less-educated households experienced losses 60% more pronounced than the general population.

These findings are critical since they provide evidence of the potential effects of the pandemic in a “best-case” scenario. More than 90% of students in The Netherlands had access to a computer at home, and more than 95% had access to the internet and a quiet place to study^7^. But even in this context of high levels of access to digital resources, equitable funding for elementary schools, and average-to-high performance prior to the pandemic, school closures have had tangible effects on learning outcomes, especially for children with disadvantaged backgrounds.

Similar studies comparing students’ performance before and after COVID have been conducted in other countries^8,9^. Most of them have found evidence of learning losses and slower rates of growth in academic abilities during the 2020–2021 school year^10–22^, while others did not find any negative effects^23–26^.

Moreover, there is strong evidence suggesting that pre-existing inequalities in education have become more pronounced. Even before the pandemic, achievement gaps across socio-economic status (SES) were evident since kindergarten and persisted across education years^27,28^. During the pandemic, students from disadvantaged backgrounds suffered longer school closures^29^ and had less access to computers and internet for schoolwork^7,30–32^. In addition, families facing financial struggles were in less favorable positions to dedicate resources and time to school activities at home^33^. As a result of these and other limitations, learning losses have been more severe for students from racial minorities^15,19,34^, with less educated parents^6,17^ or those coming from low-income households^13,14,16,19,34,35^.

Recent attempts to synthesize the literature about learning losses^8^ estimate that students have lost the equivalent of 35% of an academic year’s worth of learning. However, further data is necessary to assess the real extent to which the pandemic has impacted learning. On one hand, the data about changes in students’ performance is still very scarce, due to the limitations that remote learning imposed on school abilities to continue standardized assessments. Moreover, students from disadvantaged groups are more likely to be underrepresented^11,34,36^, both within countries and on a global scale^8^. Therefore, further evidence is needed to assess the real extent of the effects of the pandemic across different socio-economic conditions.

Teachers are a critical source of information that has not been considered enough. Teachers were at the front line of the education efforts during the pandemic and observed the impact on student learning and academic performance firsthand. While not free of biases, they are possibly the best-informed source of information about students’ abilities to benefit from these efforts, using their own previous experience as a comparison point. Critically, teachers’ observations are available across all school contexts and socio-economic strata. Therefore, they can provide insights into the effects of the pandemic that are representative of a wider variety of contexts than the ones included in a recent analysis of individual differences. Elementary school teachers more specifically, establish a unique relationship with their students, as they instruct them in multiple subjects, compared to higher education where students’ curriculum and interests are more heterogeneous, and students are often taught different subjects by different teachers. As a result, in the current context of data scarcity, elementary school teachers may be better prepared to aggregate individual student information into group-level estimates than can be accessed through survey methods.

Moreover, understanding teacher’s experiences throughout the pandemic is of critical importance for the future of education. Multiple studies have indicated that teachers have experienced higher levels of dissatisfaction and a lower sense of success during the pandemic^37–41^, resulting in increased levels of attrition rates worldwide^42^.

The present study presents the results of a survey distributed to teachers in Canada and the US, right at the end of the 2020–2021 school year. Our survey obtained participants’ assessments about three overarching issues: (1) How did teachers experience the transition to emergency remote learning? (2) How were equitable opportunities to access education impacted by school closures? and (3) Have students experienced learning losses or gains during the pandemic? We also collected additional data about variables regarding the socio-economic context of students to explore the generalizability of our data to different school and classroom contexts.

## Results

### Teachers’ experience transitioning from in-person to remote classes

Table 1 summarizes some of the variables that assessed teachers’ experience transitioning to remote learning. We expected that teachers’ previous experiences with online teaching and technology may have influenced how well they adapted to these changes. Overall, the observed distributions show that we recruited participants with different levels of previous preparation and training in both countries.

**Table 1:** Teachers report about their experience transitioning to remote instruction.

	Canada		USA		Total	
	*n*	prop	*n*	prop	*n*	prop
Previous experiance teaching online						
∘ No previous experience	243	0.54	283	0.62	526	0.58
∘ Some previous experience	140	0.31	126	0.27	266	0.29
∘ Already teaching online full-time	69	0.15	49	0.11	118	0.13
Training received before school closures						
∘ None	120	0.27	100	0.22	220	0.24
∘ Only at the beginning of school closures	123	0.27	109	0.24	232	0.26
∘ Only during the summer before the 2020-21 school year	115	0.26	114	0.25	229	0.25
∘ Both at the beginning of school closures and the summer before the 2020-21 school year	92	0.20	133	0.29	225	0.25
Self-rates of digital skills						
∘ Extremely bad	3	0.01	5	0.01	8	0.01
∘ Somewhat bad	27	0.06	32	0.07	59	0.06
∘ Neither good, nor bad	91	0.20	56	0.12	147	0.16
∘ Somewhat good	250	0.55	241	0.53	491	0.54
∘ Extremely good	82	0.18	124	0.27	206	0.23
How challenging was to switch to remote instructions						
∘ Not challenging at all	13	0.03	10	0.03	23	0.03
∘ Slightly challenging	35	0.09	38	0.10	73	0.09
∘ Moderately challenging	116	0.30	107	0.27	223	0.29
∘ Very challenging	153	0.39	144	0.37	297	0.38
∘ Extremely challenging	74	0.19	92	0.23	166	0.21
Effectiveness during online lessons						
∘ More effective teaching online	224	0.57	227	0.58	451	0.58
∘ Equally effective online or in-person	147	0.38	139	0.36	286	0.37
∘ More effective teaching in person	19	0.05	22	0.06	41	0.05
Preferences for teaching in the future						
∘ In person only	218	0.56	228	0.60	446	0.58
∘ Online and in-person combined	135	0.35	115	0.30	250	0.33
∘ Online only	26	0.07	31	0.08	57	0.07
∘ Not sure	7	0.02	6	0.02	13	0.02
Considered retiring during the pandemic						
∘ Yes	122	0.28	147	0.33	269	0.30
∘ No	318	0.72	304	0.67	622	0.70

Notably, the proportion of teachers with no previous experience teaching online goes from 40% for high-SES students, to more than 75% for low SES students. This association was statistically significant $({\tau }_{c}=0.22{;p}\, < \,0.001)$. Although weak, we also found significant interactions between student’s income level and the amount of training teachers received (*X*^2^ = 23.44; *p* = 0.024, *df* = 12, *Cramer*′*sV* = 0.09). We also observed higher levels of proficiency using digital technologies for educational purposes $({\tau }_{c}=0.08{;p}=0.007)$ for teacher of higher-income students. As we expected, switching to remote education was increasingly challenging for teachers with less experience teaching online $({\tau }_{c}=-0.18{;p}\, < \,0.001)$, and those with poor digital skills $({\tau }_{c}=-0.11{;p}\, < \,0.001)$.

### Equitable opportunities to access education

Multiple items throughout the survey assessed to what extent learning opportunities were offered to students and their ability to benefit from them (Table 2). More than 96% of participants agreed that *most* to *all* students in their classroom had access to the resources needed for online classes. The distribution of responses was slightly different between countries (*X*^2^ = 17.82, *p* < 0.001, *df* = 3, *Cramer*′*sV* = 0.15). But overall, even for teachers that had low-income students, reporting that few or none of their students had access to technology was rare.

**Table 2:** Teachers’ report of students’ access to education opportunities during remote instruction.

	Canada		USA		Total	
	*n*	prop	*n*	prop	*n*	prop
Students with access to technology						
∘ None	0	0.00	2	0.00	2	0.00
∘ Few	9	0.02	18	0.05	27	0.03
∘ Most	229	0.58	174	0.44	403	0.51
∘ All	160	0.40	204	0.51	364	0.46
Attendance to class						
∘ Lower than in-person	206	0.65	221	0.69	427	0.67
∘ Same as in-person	64	0.20	48	0.15	112	0.18
∘ Higher than in-person	46	0.15	50	0.16	96	0.15
Consistency of attendance to class						
∘ The same throughout the year	87	0.28	86	0.27	173	0.27
∘ Higher at the beginning of the year	86	0.27	56	0.17	142	0.22
∘ Higher at the end of the year	45	0.14	71	0.22	116	0.18
∘ Fluctuated throughout the year	99	0.31	107	0.34	206	0.33
Average proportions of students who…						
∘ Came to class regularly	0.72		0.68		0.70	
∘ Came to class irregularly	0.20		0.23		0.21	
∘ Were completely absent	0.08		0.09		0.09	
Content covered during class, compared to regular school years						
∘ Considerably less	74	0.17	93	0.20	167	0.18
∘ Slightly less	231	0.52	208	0.46	439	0.49
∘ Same as in-person	85	0.19	112	0.25	197	0.22
∘ Slightly more	42	0.09	29	0.06	71	0.08
∘ Considerably more	13	0.03	15	0.03	28	0.03
Adult assistance with remote classes was…						
∘ Not recommended	66	0.16	66	0.16	132	0.17
∘ Recommended	222	0.56	220	0.55	442	0.55
∘ Needed	110	0.28	114	0.29	224	0.28
Adult support was received by						
∘ None of the students	2	0.01	4	0.01	6	0.01
∘ Few students	96	0.24	129	0.32	225	0.28
∘ Most students	240	0.61	221	0.55	461	0.58
∘ All of the students	56	0.14	45	0.11	101	0.13

Despite having the means to access online education, more than 65% of participants indicated that attendance to class decreased during the 2020–2021 school year. Overall, there was no significant difference in teachers’ reports of attendance across countries (*X*^2^ = 2.97, *p* < 0.227, *df* = 2, *Cramer*′*sV* = 0.07). However, there was a difference in the association between attendance levels and students’ income across countries. For teachers in the US, lower levels of attendance were reported more frequently when students came from low-income households $({\tau }_{c}=-0.19{;p}\, < \,0.001)$. For Canadian teachers, this association was not present $({\tau }_{c}=-0.03{;p}\, < \,0.517)$.

Knowing the limitations of this survey in terms of providing individual data about attendance, we included one additional question to explore approximately what proportion of students were missing from the classroom. We asked respondents to break down their students into three different groups: students who attended regularly, students who attended irregularly and students who were completely absent from class throughout the whole year. According to teachers’ estimations, an average of 69.98% of students were present regularly in class, 21.24% came to class only irregularly and another 8.78% were completely absent during the whole school year. The proportion of students completely absent was consistently low for all SES levels $(F(4,611)=0.46,{p}=0.764,{\eta }^{2}=0.01)$. In contrast, the number of students attending regularly increased linearly with SES levels $(F(4,611)=2.41,{p}=0.048,{\eta }^{2}=0.02{;linear\; trend}:t=2.12,{SE}=3.16,{p}=0.034)$. Since these proportions are complementary, the proportion of students attending irregularly also decreased across SES levels $(F(4,611)=3.34,{p}=0.010,{\eta }^{2}=0.02{;linear\; trend}:t=-2.52,{SE}=2.28,{p}=0.012)$.

During class, most participants indicated that they covered less content during online lessons than they do in a regular school year. Moreover, around 28% of participants considered that adult assistance was needed for students to complete schoolwork. Whether the support from a parent or caregiver was imperative or not, we also asked participants to estimate, approximately, what proportion of their students received help at home. More than 70% of participants perceived that most to all students in their class had the support of an adult to some degree. But more importantly, perceived levels of support were higher for teachers of students coming from higher-income households $({\tau }_{c}=-0.25{;p}\, < \,0.001)$.

### Changes in academic performance during the pandemic

**Fig. 1:**
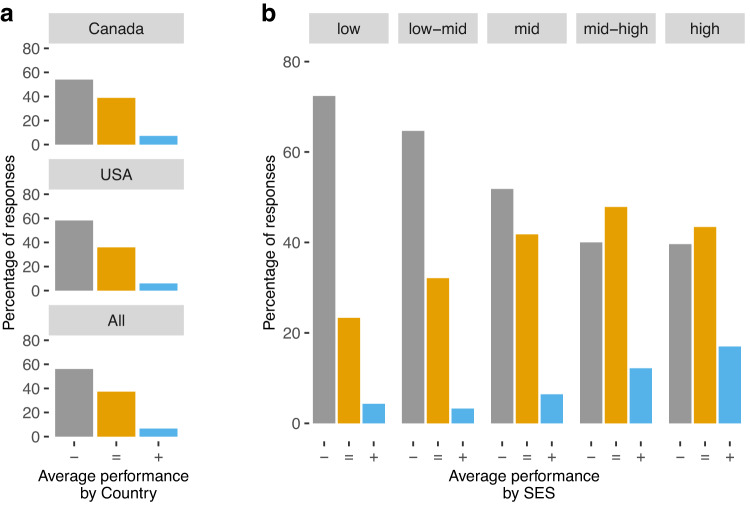
Students overall performance during the pandemic. Teachers’ perceptions of the overall performance of students, compared to a regular school year (**a**) by country and (**b**) by classroom SES. Legend: - On average, students have performed below the expectations for their grade = On average, students have performed according to the expectations for their grade + On average, students have performed above the expectations for their grade.

Another important goal of our survey was to get teachers’ input on how different aspects of academic achievement may have been affected because of the interruption of in-person classes (Table 3). More than 50% of teachers indicated that children in their class performed worse than in previous years (Fig. 1a). Moreover, teachers who reported having students from lower socio-economic status were more likely to report that performance was below the expectations for the grade (Fig. 1b; ${\tau }_{c}=-0.25{;p}\, < \,0.001$). There were no differences across countries in these estimations of students’ average performance (*X*^2^ = 2.97, *p* < 0.227, *df* = 2, *Cramer*′*sV* = 0.07).

**Table 3:** Teachers’ report of changes in academic achievement during remote instruction.

	Canada		USA		Total		
	*n*	prop	*n*	prop	*n*	prop	
Student’s overall performance during the 2020–21 school year							
∘ Below expectations for their grade	238	0.54	263	0.58	501		0.56
∘ According to expectations for their grade	171	0.39	162	0.36	333	0.37	
∘ Above expectations for their grade	32	0.07	27	0.06	59	0.07	
Academic performance by domain, compared to in-person classes							
Mathematics							
∘ Much worse	59	0.14	68	0.16	127	0.15	
∘ Somewhat worse	143	0.33	178	0.41	321	0.37	
∘ About the same	125	0.29	103	0.24	228	0.26	
∘ Somewhat better	68	0.16	48	0.11	116	0.14	
∘ Much better	32	0.08	37	0.08	69	0.08	
Reading / Literature							
∘ Much better	39	0.09	57	0.12	96	0.10	
∘ Somewhat better	136	0.31	168	0.38	304	0.34	
∘ About the same	148	0.33	125	0.28	273	0.31	
∘ Somewhat worse	84	0.19	52	0.11	136	0.15	
∘ Much worse	37	0.08	43	0.10	80	0.10	
Spelling							
∘ Much better	50	0.11	78	0.17	128	0.14	
∘ Somewhat better	140	0.32	159	0.36	299	0.34	
∘ About the same	155	0.35	121	0.27	276	0.31	
∘ Somewhat worse	59	0.13	57	0.13	116	0.13	
∘ Much worse	38	0.09	33	0.07	71	0.08	
Differences between low and high-performing students							
∘ Decreased	46	0.10	42	0.09	88	0.10	
∘ Stayed the same	130	0.30	156	0.35	286	0.32	
∘ Increased	265	0.60	254	0.56	519	0.58	
Long term effects of the pandemic on students							
∘ Lasting, negative effects	301	0.70	297	0.66	598	0.68	
∘ No lasting effects	72	0.17	79	0.18	151	0.17	
∘ Lasting, positive effects	57	0.13	73	0.16	130	0.15

Previous reports have suggested that learning losses during the pandemic have not been equally severe across different learning domains^11^. Motivated by those results, we asked participants to rate students’ performance in Math, Reading/Literature, and Spelling/Writing, separately. The distribution of responses for the three domains was slightly skewed, as most teachers reported learning losses to some degree for the three areas. We wanted to know if teachers’ perceptions of academic loss for specific domains varied depending on the subject they teach. Unfortunately, around 60% of our participants did not report that information. Moreover, out of the 40% who reported the subjects they were teaching, more than half of them taught multiple subjects that covered the three topics of interest. Nonetheless, we ran an exploratory analysis including just that 40% and we did not observe significant effects. (i.e. participants who teach math-related areas do not report better or worse learning losses in math when compared to other participants).

To complement these overall ratings, we requested more detailed information about the distribution of students in their classrooms, according to their performance level. Participants were asked to classify their students into three categories: students who performed below the expectations for their grade, students who performed according to the expectations for their grade, and students who performed above the expectations for their grade. Even though our data cannot inform about individual differences in performance, with this question we expected to obtain an estimate of the proportion of students who experienced the learning losses reported in the previous questions.

**Fig. 2:**
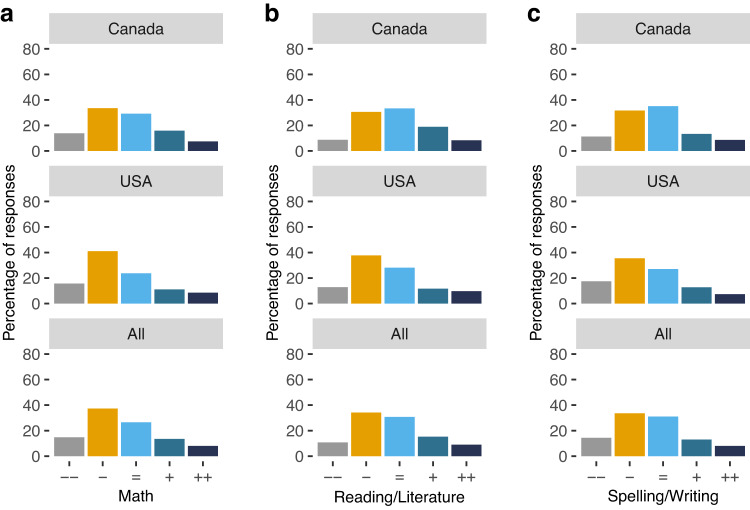
Performance across different subjects. Average performance of students compared to a regular school year in (**a**) Math, (**b**) Reading/Literature, and (**c**) Spelling/Writing. Legend: -- Much worse- Somewhat worse = About the same + Somewhat better + + Much better.

Comparing the data across the three domains did not yield significant differences in the severity of learning losses that teachers report for Math, Reading, or Spelling (Fig. 2). However, we did find differences across countries in the proportions of low, average, or high-performance students that teachers reported across all domains. Canadian teachers reported lower percentages than their US counterparts of students performing below standards during the 2020–21 school year $(F(1,904)=7.23,{p}=0.007,{\eta }^{2}=0.01)$. They also reported higher proportions of students performing above standards for their grades despite the pandemic $(F(1,905)=37.54,{p}\, < \,0.001,{\eta }^{2}=0.03)$. In summary, even though teachers of both countries reported an overall decrease in students’ performance, teachers from the US report having a higher percentage of students experiencing these losses.

Participants were also asked to estimate whether the gap between the students performing at the higher level, and those performing at the lowest level had increased, decreased, or stayed the same, compared to a typical school year. This question was designed to elicit teachers’ views of individual differences between students in their classrooms. About 58% of teachers indicated that differences between students had widened during the 2020–2021 school year, in contrast to around 32% who didn’t perceive any changes and another 10% who indicated that this gap decreased. Finally, we included one general question in the survey to ask teachers if they believed that the pandemic would have long lasting effects on students and, if so, whether these effects would have a positive or negative outcome. A large proportion of the participants expressed that the changes occurring during the pandemic would most likely have a negative impact on students’ learning in the long run.

## Discussion

We distributed a survey to primary school teachers in the US and Canada at the end of the 2020–2021 school year. Our survey was able to reach teachers from different levels of SES, who were affected by school closure at varying degrees. Their responses provided relevant insights into how education took place during the COVID-19 health crisis, especially during the 2020–2021 school year, the first to fully occur within the pandemic.

Results from our survey suggest that a large proportion of students in both countries had access to the digital resources required to access these online alternatives (such as computers, internet, etc.). This was especially true for students from advantaged homes, but even in the lower SES levels, more than 90% of students had access to digital resources. This is not surprising, considering recent statistics showing that around 93% and 88% of students in Canada and the US, respectively, have access to a computer at home and more than 95% have access to the internet in both countries^7,32^.

However, the availability of digital resources is necessary but not sufficient to guarantee that students have access to educational opportunities. For example, our data indicates that the amount of instruction time decreased substantially, compared to a regular school year. Instruction time requirements for primary school in both Canada^43^ and the US^44^ vary across states, but the average is close to 30 h per week. The average number of hours of remote instruction reported by our participants fell below the 20 h, which represents less than two thirds of these typical requirements. Consistently, most participants reported not being able to deliver all the content they typically taught during a regular school year. In addition, most participants indicated that attendance to class was lower than in a traditional year. Was this trend due to just a few, or to many students consistently missing class? On average, our respondents report that approximately 3 in every 10 students in their class were attending inconsistently or completely absent. Although small, the reported proportions of students who were completely absent from class are of critical importance, since they represent students who were not able to benefit from education opportunities at all during the last school year.

Overall, nearly 56% of our participants agreed that students performed below the expectations for their grades during the 2020–2021 school year. These reports are converging with previous studies using standardized tests to compare students’ academic achievement before and during the pandemic (Engzell et al., 2020; Kuhfeld et al., 2020). Unlike previous studies, teachers’ rates of academic performance obtained during our survey do not suggest that the drop in math performance was more pronounced than in other domains (i.e., reading). It is possible that differences between learning losses experienced across domains exist in our student population, as suggested by studies analyzing individual data on standardized tests. However, those differences may not be large enough to be captured by the limited response options presented in our survey. It is also possible that presenting this question in a grid format may have increased the probability of straight-lining, or the tendency in which participants select the same answer choice to all items on the question.

More importantly, teachers’ rates of academic performance varied drastically according to the income-level of their students, and more than half of our participants agreed that differences between low and high performing students became more pronounced during the 2020–2021 school year. This learning gap between low and high performing students is fundamentally different from the overall performance trend. Assuming that teachers’ ratings are an accurate depiction of how actual performance was impacted by the school closures, the questions about overall performance should reflect perceived changes on the mean of the distribution, whereas the questions about the learning gap should reflect perceived changes on the difference between the lower and the upper tail of the distribution within their classrooms.

**Fig. 3:**
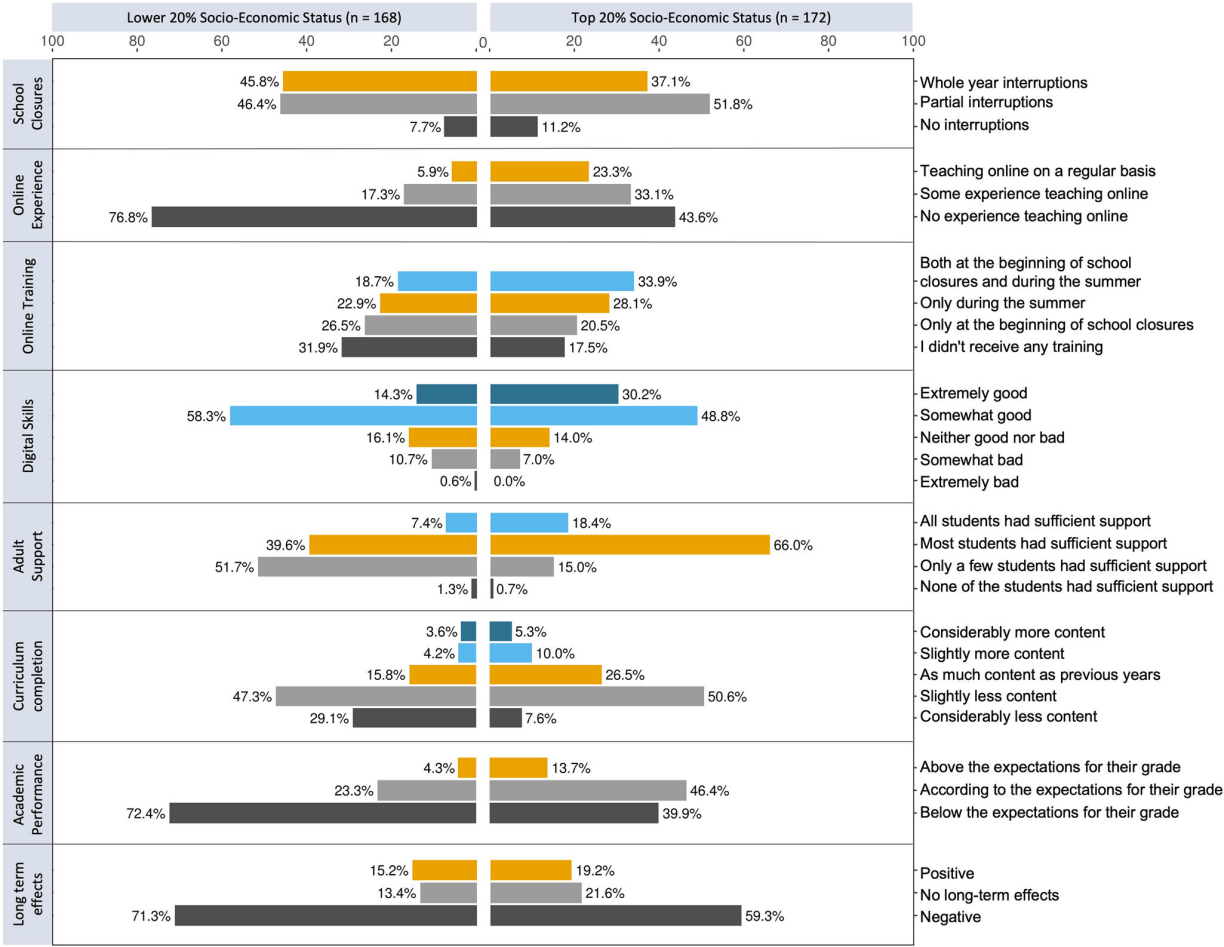
Comparison between students on top and bottom 20% of socio-economic status. We created two groups to represent the extremes of the SES distribution. To make the groups comparable in terms of size, the lower SES group included participants who reported that their students come from predominantly Low-Income households (*n* = 168), whereas the higher SES group included participants whose students predominantly come from High-Income households (*n* = 53), or a mix of Middle and High-Income (*n* = 119). Since our perceived SES measure is on a discrete scale, selecting exactly the top and bottom 20% is not possible. Instead, the lower and higher income groups represent 18.44% and 18.88% of the distribution.

Like previous studies in the literature, our findings suggest that the pandemic has emphasized individual differences between students of different income levels, that are otherwise attenuated during in-person instruction. Figure 3 highlights the most noticeable differences between the lower and the top 20% of the SES distribution. The consistent pattern of interaction between teachers’ reports of the effects of the pandemic and their students’ socio-economic background suggests that students from low- and high-income households may have experienced school closures in very different ways.

First, our data suggest that teachers from classrooms with higher income levels may have been more prepared for the transition to remote alternatives, as they had more relevant experience with online instruction before the pandemic and they had better self-ratings of digital skills than teachers from lower SES classrooms. For example, 7 out of every 10 teachers of students in the lower 20% of the SES were teaching online for the first time during the pandemic, versus only 4 out of every 10 in the top 20% SES.

During the school closures, teachers from higher SES classrooms were also less likely to report a drop in overall attendance levels to online lessons, compared to a regular school year, and had higher proportions of students who consistently attended class. Moreover, they observed students receiving support from adults at home more frequently. This was one of the most striking contrasts observed in our data, which became more evident when comparing the two extremes of the distribution. Taken together, these results suggest that students in higher income levels may have been in a better position to benefit from the remote alternatives offered during the pandemic. Consistent with this prediction, teachers from higher income classrooms were also less likely to report learning losses during the pandemic.

These results have critical implications for our understanding of the long-term effect of the pandemic. Household income was already an important predictor of future academic achievement before the pandemic. With the closure of schools as a measure to contain the spread of the COVID-19 virus, children from disadvantaged socio-economic backgrounds who were already in a vulnerable position may find themselves falling further behind their peers. As a result, they may be more likely to experience dropout in the future and less likely to pursue higher levels of education, which may reinforce the already existing income inequalities into future generations.

There are limitations to our results due to the observational nature of the data. It is possible that some of the associations observed are the results of biases in teachers’ perceptions. In addition, it is important to bear in mind that teacher reports offer information that occurs at the classroom level and therefore cannot account for effects at the individual level.

Despite these limitations, teachers can provide insights into the effects that the pandemic has had on students that is unique and highly valuable. Teachers have been active observers of students’ performance before, during, and after the pandemic. They receive a constant stream of data from students and therefore may perceive trends that standardized tests taken at a single time point may not capture.

In addition, teachers can provide information that is representative of a wide range of socio-economic and classroom contexts, something that has been a limitation of previous analyses of individual data. Our survey has its own limitations when describing the effects of SES on learning during the pandemic. For instance, we cannot guarantee that the SES levels reported by teachers in the US will correspond perfectly with the same levels in Canada. In other words, what teachers consider low SES in one country may be considered middle SES in the other. But even if the levels do not overlap perfectly, what seems to be consistent across our data is that students in lower levels struggled more during the pandemic and that trend remains when analyses are conducted on each country separately.

Critically, the relevance of teacher surveys is not only limited to their role as informants of students’ achievement. Teachers have a critical role in carrying forward education efforts and understanding how they experienced the recent crisis is by itself a critical question that current research should address. The stress associated with abrupt changes in the work environment, combined with the high demands and responsibility levels puts teachers at risk of experiencing work-related burnout. In fact, previous studies have found that, during 2020, teachers were more likely to consider leaving the classroom before retirement age^39,45–47^, and at least 23% considered retiring specifically due to the pandemic^48^, which has aggravated the already existing global crisis of teacher shortages^42^. In our survey, as expected, the frequency of teachers considering leaving their profession was higher for those with more years of experience. However, even in the group of less experienced teachers, around 1 in every 4 considered retiring during the pandemic. Teachers are expected to continue to have a critical role as the pandemic continues to unfold and in future efforts to mitigate the learning losses experienced by students during this period. It is evident from these results that understanding teachers’ experiences and providing them with the necessary resources and support will be critical for the success of these efforts.

In summary, our results provide an insight into how teachers from these countries experienced remote education, and their observations about consequences for students’ academic achievement, measured right at the end of the first school year to fully occur amidst the pandemic. Our sample was diverse in terms of the geographical distribution of responses and the socio-economic background of the students. Nevertheless, our results may be specific to the higher-level socio-economic characteristics of these countries and may not be generalizable to different contexts. Our results suggest that even in the presence of widespread access to digital learning tools, consistent attendance to class and complete delivery of the curriculum could not be guaranteed. Most teachers reported observing a decline in students’ academic performance, and a growth in the gaps between low and high performing students. More importantly, our data suggest that the effects of the pandemic were not equally distributed. Students from lower SES levels had teachers who were less prepared for the transition to online activities and received less support from adults during homeschooling. Consistently, teachers from lower SES classrooms also reported drops in performance more frequently than those from the higher SES levels.

Even though the group estimations that teachers provide at the classroom level are not enough to suggest causal relationships between the variables we studied and individual differences in academic achievement, teachers contribute valuable information, based on their constant interaction with students. Their observations provide a unique perspective on the effects of the pandemic that is relevant to inform policy decisions and future research.

## Methods

### Participants

**Fig. 4:**
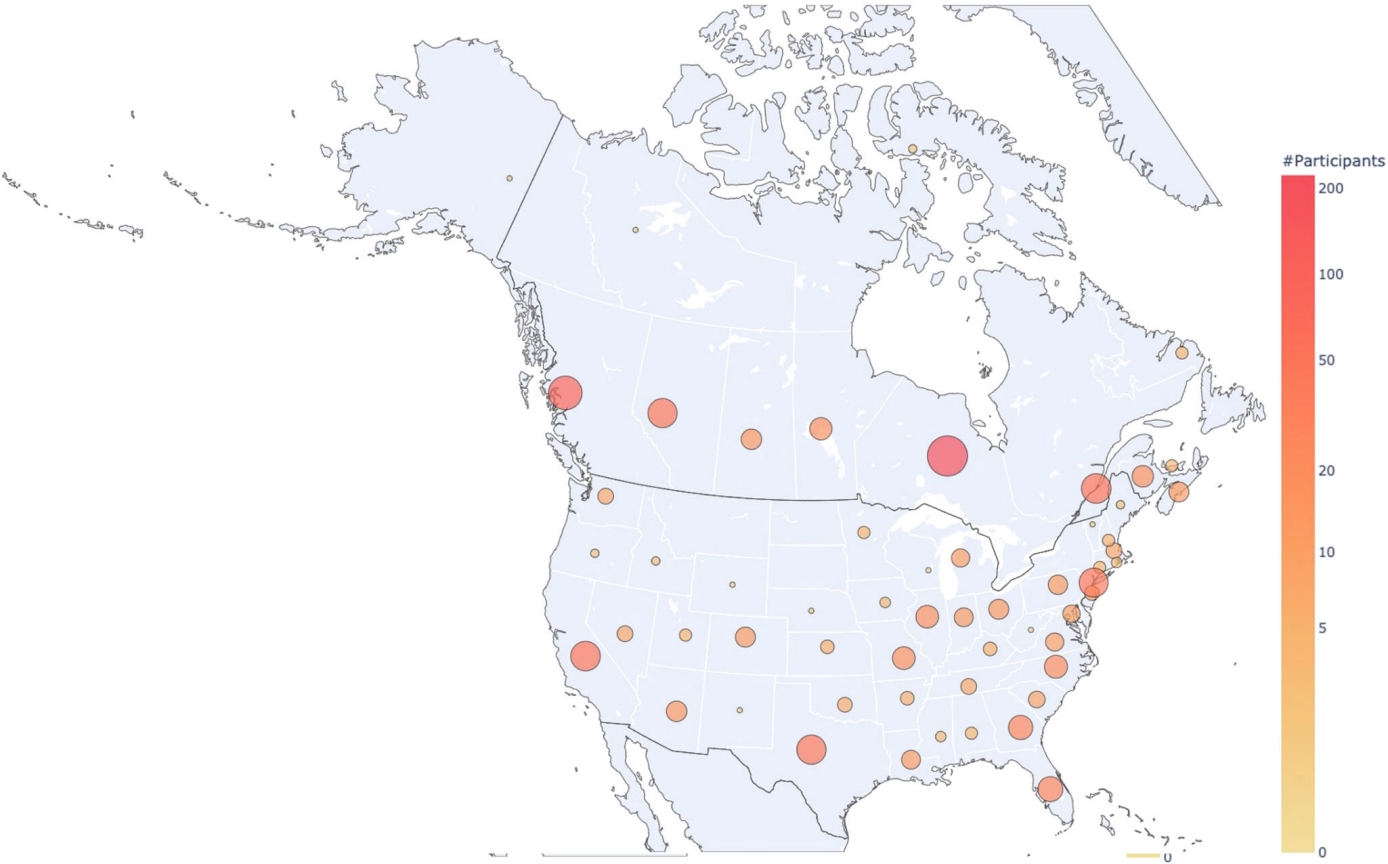
Geographical distribution of participants. Distribution of responses collected across Canada and the US^49^ The circle size represents the amount of participants recruited, transformed to log scale.

Teachers from public elementary schools were recruited through the Qualtrics Online Sample panel. We aimed at a sample size of 900 participants, 450 from Canada and 450 from the US. Our sample size was constrained by the availability of participants from the Qualtrics panel that fit into our inclusion criteria. We required participants to be elementary school teachers (grades 1 to 6), fluent in English, living in Canada or the US, who were actively teaching during the 2020–2021 school year. We surveyed 918 participants between June 16th and June 28th, 2021. Seven participants were removed for having a large number of missing responses. The final sample included 911 participants, 453 from Canada and 458 from the US (Fig. 4). The complete dataset can be accessed here: https://osf.io/3dsef.

Our sample was diverse in terms of the professional background of participants and the socioeconomic characteristics of their students (see Table 4). We did not consider participants’ socioeconomic status (SES) when determining inclusion. In fact, we were not able to select participants across specific SES levels since the Qualtrics Online Sample of teachers was already limited. Rather, we recruited all potential participants and subsequently described the income level of the students they teach, as reported by the participants themselves.

**Table 4:** Socio-demographic characteristics of participants.

	Canada		USA		Total	
	*n*	prop	*n*	prop	*n*	prop
Education						
∘ <High-School	0	0.0	1	0.0	1	0.00
∘ High-School	5	0.01	9	0.02	14	0.02
∘ College (2 years)	33	0.07	32	0.07	65	0.07
∘ College (4 years)	248	0.55	217	0.48	465	0.51
∘ Master’s	155	0.35	188	0.41	343	0.38
∘ Ph.D.	8	0.02	9	0.02	17	0.02
Employment status						
∘ Part-time (<50% full-time)	8	0.02	8	0.02	16	0.02
∘ Part-time (51–70% full-time)	17	0.04	14	0.03	31	0.03
∘ Part-time (71–90% full-time)	51	0.11	32	0.07	83	0.09
∘ Full-time (>90% full time)	374	0.83	404	0.88	778	0.86
Special education teachers						
∘ Yes	72	0.16	120	0.26	192	0.21
∘ No	366	0.84	338	0.74	704	0.79
Student socio-economic background						
∘ Low-income	38	0.09	130	0.29	168	0.19
∘ Mix of low- and middle-income	105	0.24	113	0.25	218	0.25
∘ Middle-income	203	0.47	127	0.28	330	0.37
∘ Mix of middle- and high-income	56	0.13	63	0.14	119	0.13
∘ High-income	33	0.07	20	0.04	53	0.06
Grades participants were teaching during the 2020-2021 school year						
∘ 1	44	0.10	53	0.12	97	0.11
∘ 2	44	0.10	40	0.09	84	0.09
∘ 3	56	0.12	47	0.10	103	0.11
∘ 4	61	0.13	57	0.12	118	0.13
∘ 5	59	0.13	50	0.11	109	0.12
∘ 6	49	0.11	36	0.08	85	0.09
∘ Multiple grades	140	0.31	175	0.38	315	0.35
Teaching experience						
Mean (years)	10.05		11.81		10.95	
SD	7.34		9.52		8.56	

There were small differences between participants of both countries. For example, teachers from the US were on average more experienced than their Canadian counterparts (*X*_*Can*_ = 10.05 years, *X*_*USA*_ = 11.82 years; *t*(822.63) = −2.14, *p* = 0.033, *d* = 0.14) and reported having students from lower-income households to a greater extent (*X*^2^ = 71.44, *p* = 0.000, *df* = 4, *Cramer*′*sV* = 0.20).

More than 90% of teachers in our sample experienced school closures during the pandemic, ranging from a few days to the whole year (Table 5). Partial closures were, on average, larger in Canada compared to the US (*t*(409.40) = 3.32, *p* = 0.001, *d* = 0.33). During remote instruction, participants reported spending around 18.87 h of class time per week. Furthermore, most participants received classwork from students on a weekly or daily basis and provided feedback with similar frequency. These survey items offered an estimate of the amount of information that participants received from students, which will serve as a basis for their judgments about academic performance.

**Table 5:** Teachers’ reports of the length of interruptions to in-person classes they experienced and alternatives offered to students.

	Canada		USA		Total	
	*n*	prop	*n*	prop	*n*	prop
Interruption of in-person classes						
∘ Never interrupted	31	0.07	40	0.09	71	0.08
∘ Partially interrupted	254	0.57	245	0.53	499	0.55
∘ Interrupted during the whole year	159	0.36	172	0.38	331	0.37
Length of partial interruptions						
Mean (days)	90		74		82	
SD	51		56		55	
Alternative instruction method implemented during school closures^1^						
∘ Online, synchronous	317	0.70	320	0.70	637	0.70
∘ Online, asynchronous	180	0.40	206	0.45	386	0.42
∘ Remote, other media	40	0.09	53	0.12	93	0.10
∘ None of the above	15	0.03	18	0.04	33	0.04

^1^Multiple choice question, proportions do not add to 1. Except for 12 participants (1.32% of the whole sample), remote alternatives with other media were always offered in combination with online lessons.

Since most of the observed discrepancies between countries corresponded to small effect sizes, we considered both groups of participants to be comparable. Therefore, we report here the results corresponding to the whole sample.

### Procedure

The study was approved by the Non-medical Research Ethics Board of the University of Western Ontario. We administered the survey through the Qualtrics online platform. All the participants on the Qualtrics panel who potentially met our inclusion criteria received an email with a link to the survey and the estimated time commitment. Participants who accessed the link were presented with the letter of information (LOI) before starting the survey. Since the survey was administered online, participants could not provide written consent. Instead, they indicated agreement to participate by ticking a checkbox at the end of the LOI. The survey was presented only to those participants who provided this type of consent.

We asked participants to complete the survey in a single session, which should have taken approximately 10 min. To minimize the risk of missing data, we required responses for most survey items. However, all the questions with response requirements included an ‘I prefer not to answer’ option that participants could use if they didn’t feel comfortable disclosing the required information. The complete survey is available here: https://osf.io/bx63k/.

### Reporting summary

Further information on research design is available in the Nature Research Reporting Summary linked to this article.

### Supplementary information

Reporting Summary

## Data Availability

The data that support the findings of this study are openly available in the Open Science Framework at https://osf.io/3dsef.
