# Supplementary material for: Elementary school teachers’ perspectives about learning during the COVID-19 pandemic
Source: NPJ Sci Learn. 2023 Sep 18;8:40. doi: 10.1038/s41539-023-00191-w (PMC10507123; doi:10.1038/s41539-023-00191-w)
Supplement: Supplementary file 1 — Reporting Summary [file 41539_2023_191_MOESM1_ESM.pdf]

nature portfolio

Corresponding author(s): Aymee Alvarez-Rivero

Last updated by author(s): Aug 21, 2023

Reporting Summary

Nature Portfolio wishes to improve the reproducibility of the work that we publish. This form provides structure for consistency and transparency in reporting. For further information on Nature Portfolio policies, see our Editorial Policies and the Editorial Policy Checklist.

Statistics

For all statistical analyses, confirm that the following items are present in the figure legend, table legend, main text, or Methods section.

n/a	Confirmed	
☐	☒	The exact sample size (*n*) for each experimental group/condition, given as a discrete number and unit of measurement
☐	☒	A statement on whether measurements were taken from distinct samples or whether the same sample was measured repeatedly
☐	☒	The statistical test(s) used AND whether they are one- or two-sided<br>*Only common tests should be described solely by name; describe more complex techniques in the Methods section.*
☐	☒	A description of all covariates tested
☐	☒	A description of any assumptions or corrections, such as tests of normality and adjustment for multiple comparisons
☐	☒	A full description of the statistical parameters including central tendency (e.g. means) or other basic estimates (e.g. regression coefficient) AND variation (e.g. standard deviation) or associated estimates of uncertainty (e.g. confidence intervals)
☐	☒	For null hypothesis testing, the test statistic (e.g. *F*, *t*, *r*) with confidence intervals, effect sizes, degrees of freedom and *P* value noted<br>*Give P values as exact values whenever suitable.*
☒	☐	For Bayesian analysis, information on the choice of priors and Markov chain Monte Carlo settings
☒	☐	For hierarchical and complex designs, identification of the appropriate level for tests and full reporting of outcomes
☐	☒	Estimates of effect sizes (e.g. Cohen's *d*, Pearson's *r*), indicating how they were calculated

Our web collection on statistics for biologists contains articles on many of the points above.

Software and code

Policy information about availability of computer code

Data collection	All data collection ocurred on Qualtrics online platform (https://www.qualtrics.com/)
Data analysis	Data was analyzed using R (version 4.1,1) on RStudio (1.4.1717)

For manuscripts utilizing custom algorithms or software that are central to the research but not yet described in published literature, software must be made available to editors and reviewers. We strongly encourage code deposition in a community repository (e.g. GitHub). See the Nature Portfolio guidelines for submitting code & software for further information.

Data

Policy information about availability of data

All manuscripts must include a data availability statement. This statement should provide the following information, where applicable:

- Accession codes, unique identifiers, or web links for publicly available datasets
- A description of any restrictions on data availability
- For clinical datasets or third party data, please ensure that the statement adheres to our policy

The data that support the findings of this study are openly available in the Open Science Framework at https://osf.io/3dsef.

Research involving human participants, their data, or biological material

Policy information about studies with human participants or human data. See also policy information about sex, gender (identity/presentation), and sexual orientation and race, ethnicity and racism.

Reporting on sex and gender	Neither sex nor gender was used in the study design
Reporting on race, ethnicity, or other socially relevant groupings	Our participants were elementary-school teachers. We did not collect data about participants' race, ethnicity or socio-economic level. However, we did asked teachers to report an estimation of their students' household income level. Participants provided that information by answering the following survey question: "Thinking about students in your class, what type of household would you say they predominantly come from?". Answer choices were: "Low-income households", "Middle-income households", "High-income households", "A mix of low-and middle-income households", "A mix of middle-and high-income households", "I don't know", "I prefer not to answer".
Population characteristics	See above
Recruitment	Participants were recruited through Qualtrics online sample panel. We recruited all participants that fit our inclusion criteria (elementary school teachers, fluent in English, teaching during the pandemic). In other words, we did not include any systematic selection procedure other than being part of our population on interest. Although we are are not aware of any systematic biases, there is a chance that participants in this sample panel differ from the overall population in some aspects. However, we don't think there are any systematic biases on the variables that are relevant to our studies.
Ethics oversight	The study was approved by the Non-medical Research Ethics board of the University of Western Ontario

Note that full information on the approval of the study protocol must also be provided in the manuscript.

Field-specific reporting

Please select the one below that is the best fit for your research. If you are not sure, read the appropriate sections before making your selection.

☐ Life sciences ☒ Behavioural & social sciences ☐ Ecological, evolutionary & environmental sciences

For a reference copy of the document with all sections, see nature.com/documents/nr-reporting-summary-flat.pdf

Behavioural & social sciences study design

All studies must disclose on these points even when the disclosure is negative.

Study description	Survey data, analyzed using quantitative research methods
Research sample	A total of 918 Elementary school teachers (grades 1 to 6), fluent in English, living in Canada or the US, who were actively teaching during the 2020-2021 school year. Age and age was not collected. This sample was relevant for our research questions, which were concerned about the impact of the pandemic on elementary school students and teachers.
Sampling strategy	Convenience sample (Qualtrics Online Sample Panel). In order to optimize our sample size, we recruited all the participants within Qualtrics that fit our inclusion criteria.
Data collection	The survey was completed online, within the Qualtrics online platform. Therefore, researchers were not present at the moment of data collection. All the participants on the Qualtrics panel that potentially meet our inclusion criteria received an email with a link to the survey and the estimated time commitment. The survey was presented only to those participants who provided consent.
Timing	Data collection took place between June 16th and June 28th, 2021
Data exclusions	Seven participants were removed for having a large number of missing responses.
Non-participation	No participants declines participation
Randomization	Participants were not allocated into experimental groups

Reporting for specific materials, systems and methods

We require information from authors about some types of materials, experimental systems and methods used in many studies. Here, indicate whether each material, system or method listed is relevant to your study. If you are not sure if a list item applies to your research, read the appropriate section before selecting a response.

Materials & experimental systems

n/a	Involved in the study
☒	☐ Antibodies
☒	☐ Eukaryotic cell lines
☒	☐ Palaeontology and archaeology
☒	☐ Animals and other organisms
☒	☐ Clinical data
☒	☐ Dual use research of concern
☒	☐ Plants

Methods

n/a	Involved in the study
☒	☐ ChIP-seq
☒	☐ Flow cytometry
☒	☐ MRI-based neuroimaging
